# Difference in modes of firing rate modulation between cortical areas

**DOI:** 10.1186/1471-2202-14-S1-P359

**Published:** 2013-07-08

**Authors:** Yasuhiro Mochizuki, Shigeru Shinomoto

**Affiliations:** 1 Department of Physics, Kyoto University, Kyoto, 606-8502, Japan

## 

Neurons *in vivo *exhibit irregular firing, and some aspect of their firing pattern such as the degree of firing irregularity does not fluctuate greatly in time for individual neurons, while it differs among neurons [1]. By analyzing spike trains recorded from a variety of cortical areas, it has been revealed that neurons in motor-related cortical areas fire regularly while neurons in visual and prefrontal cortex fire randomly [2]. Here, the firing irregularity is defined as a variability of consecutive interspike intervals, independent of the firing rate. In those studies, the firing rate has been ignored, because it may fluctuate greatly in time depending on external stimuli or behavioral contexts of an animal.

Though the neuronal firing rate may fluctuate with external stimuli, this does not necessarily mean that the rate is extrinsic and perfectly controllable; there could be some uncontrollable intrinsic aspect in the firing rate fluctuation. In this contribution, we go contrary to the above-mentioned studies, and attempt to characterize neuronal spike trains solely in terms of the firing rate, ignoring the firing irregularity. The key feature of neuronal spike trains we now pay attention to is dynamic mode of rate fluctuation, such that the rate either fluctuates smoothly in time or changes abruptly between several stationary states.

We compare two different models of a fluctuating rate process for their goodness-of-fit with respect to spike trains recorded from single neurons in several cortical areas of rats. The first model is the state space model constructed under the Empirical Bayes method [3,4]. The generative inhomogeneous Poisson process is defined as the conditional distribution of the spike times $\left\{t_{i}\right\}\equiv \left\{t_{1},t_{2},...,t_{n}\right\}$ given the rateλ(t), $p\left(\left\{t_{i}\right\}\left|\lambda \left(t\right)\right)\right)=\prod_{i=1}^{n}\lambda \left(t_{i}\right)\text{exp}\left[-\int_{0}^{T}\lambda \left(t\right)dt\right]$. We give the prior distribution of λ(t) by incorporating the tendency of the rate to be flat, $p\left(\lambda \left(t\right)\right)\propto \text{exp}\left[-\beta \int_{0}^{T}{\left(d\lambda /dt\right)}^{2}dt\right]$. The hyperparameter β is selected by the principle of maximizing the marginal likelihood, and then we obtain the maximum *a posteriori *(MAP) estimate for the rate $\overset{⌢}{\lambda }\left(t\right)$. The second alternative model is the Hidden Markov model (HMM) whose hidden states represent different rate states [5-7]. The number of states is selected with a variation Bayes method.

In this way, two different estimations of the firing rate are obtained using the continuous state space model and the discrete HMM. We compare them for each spike train obtained from a neuron, and examine whether the continuous or discrete representation of the state transition is more plausible. Then, we examine how the different preferences are distributed in different cortical areas.
